# Surgical Orthodontic Treatment for Skeletal Maxillary Protrusion in Sturge-Weber Syndrome: A Case Report and Review of the Literature

**DOI:** 10.7759/cureus.59964

**Published:** 2024-05-09

**Authors:** Isamu Kado, Shintaro Ogashira, Shigehiro Ono, Koichi Koizumi, Takayuki Nakagawa, Yuki Yoshimi, Ryo Kunimatsu, Shota Ito, Yuma Koizumi, Tomohiro Ogasawara, Tomonao Aikawa, Kotaro Tanimoto

**Affiliations:** 1 Department of Orthodontics and Craniofacial Developmental Biology, Graduate School of Biomedical and Health Sciences, Hiroshima University, Hiroshima, JPN; 2 Department of Oral and Maxillofacial Surgery, Hiromashi University Hospital, Hiroshima, JPN; 3 Department of Oral Oncology, Graduate School of Biomedical and Health Sciences, Hiroshima University, Hiroshima, JPN; 4 Department of Oral and Maxillofacial Surgery, Graduate School of Biomedical and Health Sciences, Hiroshima University, Hiroshima, JPN

**Keywords:** sagittal split ramus osteotomy, skeletal maxillary protrusion, hemangioma, orthodontic treatment, sturge-weber syndrome

## Abstract

Sturge-Weber syndrome (SWS) is characterized by hemangiomas, glaucoma, and central nervous system disorders. Here, we report the case of a 15-year-old boy with SWS and upper-lip hypertrophy who underwent surgical orthodontic treatment for correction of a large overjet and deep overbite. In addition to the a large overjet and deep overbite, interdental spacing was observed in both the arches. The mandible was retrognathic and deviated to the right side. No maxillary occlusal canting or temporomandibular joint symptoms were observed. The patient was diagnosed with skeletal maxillary protrusion with spaced dentition and mandibular deviation to the right due to SWS. After presurgical orthodontic treatment using a multibracket appliance, we performed a sagittal split ramus osteotomy (SSRO) alone due to the presence of a hemangioma around the maxilla. No abnormal bleeding or cerebral hemorrhage due to increased blood pressure was observed during the SSRO. Postoperatively, the maxillary and mandibular arches were well-aligned, the deep overbite and excessive overjet improved, and bilateral angle class I molar and canine relationships were established. Furthermore, mandibular deviation improved, and the midlines of both arches approximately coincided with the facial midline. In conclusion, orthognathic surgery is feasible in patients with SWS after carefully evaluating the sites and sizes of the hemangiomas.

## Introduction

Sturge-Weber syndrome (SWS) is a congenital disorder characterized by capillary and vascular malformations involving facial hard and soft tissues, with a reported incidence of 1/20,000-1/50,000 [1,2]. SWS was first reported by Schirmer in 1860 and later extensively described from ophthalmological, neurological, and radiological perspectives by Sturge and Weber [3]. SWS is caused by a somatic activating mutation in the *GNAQ *gene, and it is recognized as a non-hereditary congenital disorder [4]. According to the Roach scale, SWS is classified as type I when it presents with both facial hemangioma and pial (leptomeningeal) angioma, type II when only facial hemangioma is present, and type III when only pial angioma is present [5].

The classic triad of symptoms of SWS includes skin lesions, ocular lesions, and central nervous system disorders. The main skin manifestation is a port-wine stain that often appears on the neck and head [6]. Erythema is present at birth and is caused by capillary malformations. Skin lesions are present in approximately 90% of the patients with SWS [7], are mostly distributed along the 1st and 2nd branches of the trigeminal nerve, and occur both unilaterally and bilaterally. Central nervous system symptoms include epileptic seizures, contralateral hemiplegia, and mental retardation owing to atrophy and calcification of the cerebral hemisphere. Approximately half of the patients with SWS are reported to have mental retardation [8], and epilepsy from infancy is observed in approximately 80% of patients with SWS. Ocular symptoms include glaucoma, which can eventually lead to blindness.

The most notable perioral characteristics of SWS are hemangiomas in the craniofacial region and gingival hyperplasia caused by antiepileptic drugs [1,9]. Lip malformations and hypertrophy caused by hemangiomas can cause functional impairments in speech, chewing, and swallowing. Local venous hypertension leads to hypertrophy of the facial skin and bones, resulting in dysfunction or malocclusion [10]. These deformities and dysfunctions may affect social development and could be a severe psychological burden, affecting the quality of life [10]. Moreover, hemostasis is extremely important when performing surgical procedures under these conditions. Therefore, the use of orthodontic anchor screws (OAS) and surgical treatment should be avoided in patients with SWS [11]. Reports on orthodontic treatment for patients with SWS are limited, and few studies have reported on orthognathic surgery in these patients [10]. Although surgical procedures such as ophthalmic surgery and hemispherectomy for controlling epilepsy have been reported in patients with SWS [12,13], considering the perioperative risks, surgical treatment of facial deformities is challenging in patients with SWS [10]. We describe our experience with orthognathic surgery for skeletal maxillary protrusions in a patient with SWS.

## Case presentation

Pretreatment evaluation

**Figure 1:**
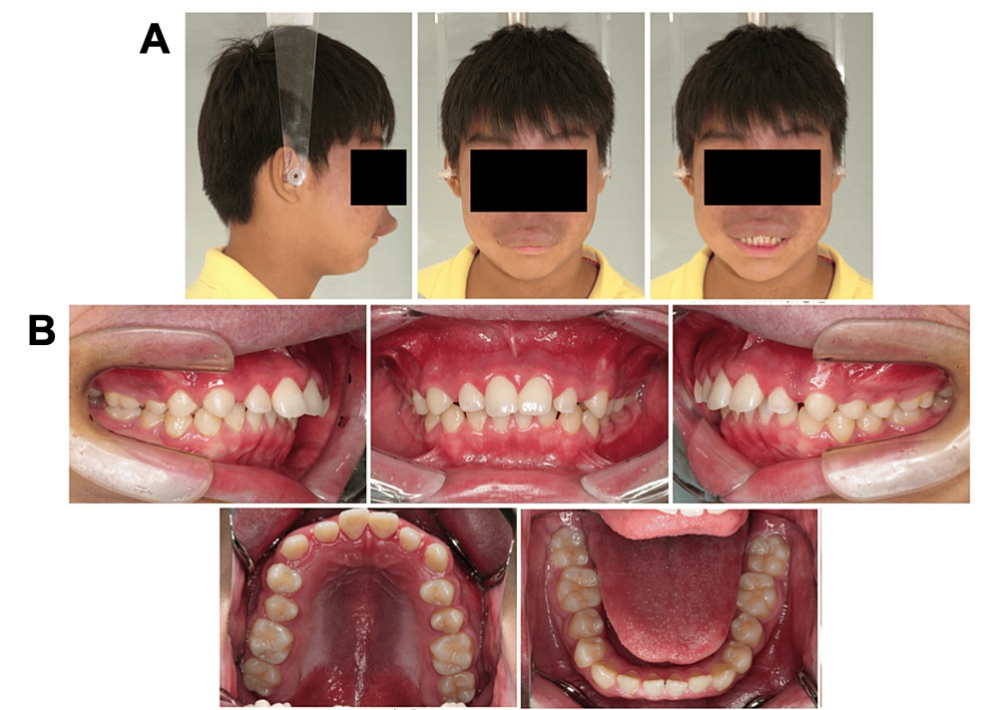
Pretreatment evaluation (A) Facial photographs. (B) Intraoral photographs.

A 15-year-old boy with chief complaints of a large overjet and protruding upper lip was referred to the Department of Orthodontics at Hiroshima University Hospital. Extraoral examination revealed a convex profile with a hypertrophic upper lip due to hemangioma, and the frontal view revealed asymmetric facial features with a mandibular shift to the right side (Figures 1A, 1B).

**Figure 2:**
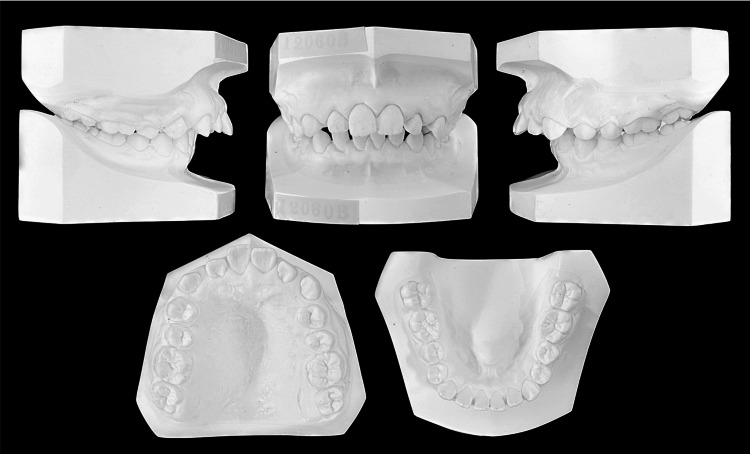
Pretreatment evaluation, Plaster models.

**Figure 3:**
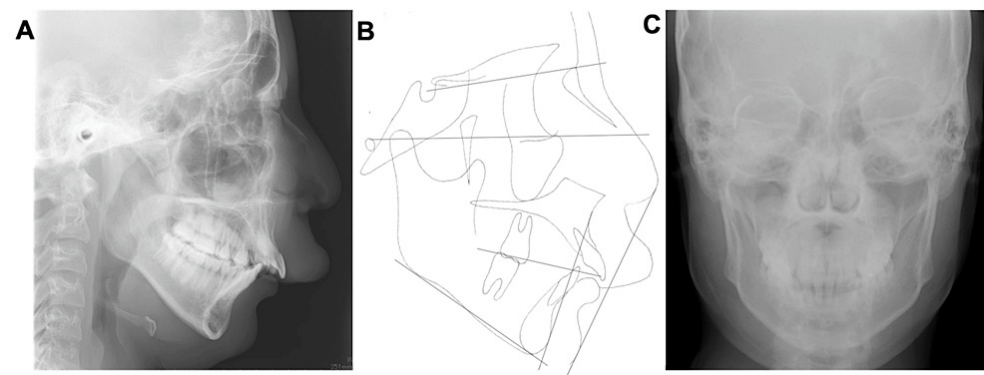
Pretreatment evaluation. (A) Lateral cephalometric radiograph. (B) Original tracing of lateral view. (C) Frontal Cephalometric radiograph.

Intraoral examination revealed interdental spacing in the upper and lower jaws. The molar relationship was full-cusp class II on the right side and end-to-end class II on the left side due to retrognathia and a right-sided mandibular shift (Figures 2, 3).

**Figure 4:**
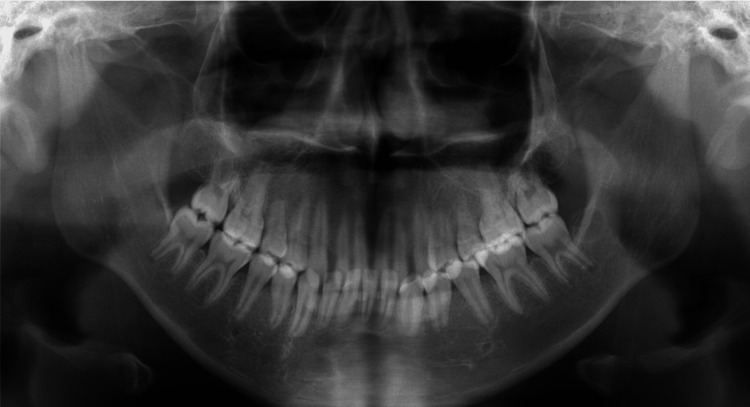
Pretreatment evaluation, Panoramic radiograph.

The midline of the upper arch coincided with the facial midline; however, the midline of the lower arch was deviated to the right by approximately 1.5 mm. No deviations were observed in the maxillary occlusal cant. The patient had an excessive overjet (13.9 mm) due to mandibular retrognathia and labial inclination of the upper incisors, and a deep overbite (4.0 mm) due to an accentuated curve of Spee. The maxillary gingiva and alveolar mucosa were swollen due to hemangiomas. Formation of the mandibular left third-molar tooth germ and normal alveolar bone level were observed on the panoramic radiograph (Figure 4).

**Figure 5:**
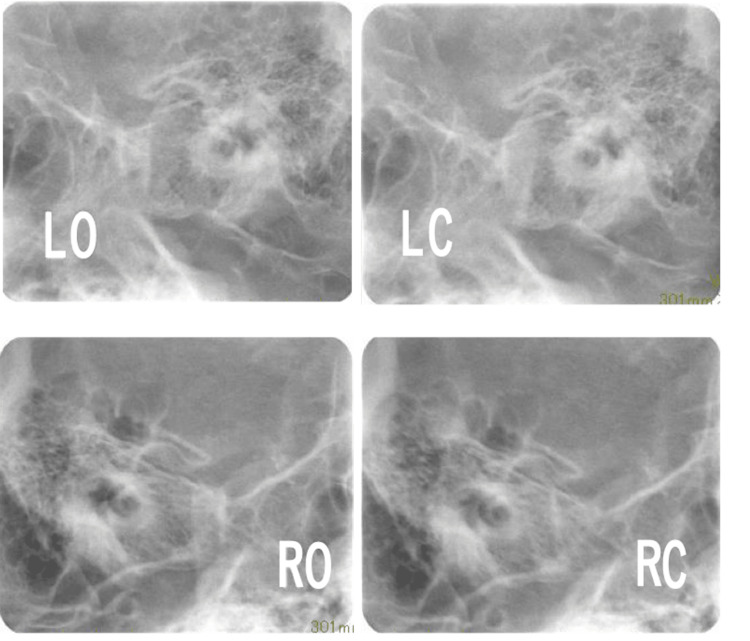
Pretreatment evaluation. Schuller’s radiographs. LO, left open; LC, left close; RO, right open; RC, right close.

Lateral cephalometric analysis revealed a skeletal Class II relationship with mandibular retrognathia and a high mandibular plane angle (Angle between NA (Nasion to point A) and NB (Nasion to point B) lines (ANB): 10.0°; Angle between NA (Nasion to point A) and NB (Nasion to point B) lines (SNA): 84.3°; Angle between the anterior cranial base (Sella to Nasion) and the NB (Nasion to point B) line (SNB): 74.3°; Angle between the FH plane (Porion to Orbitale) and the mandibular plane (Gonion to Menton) (FMA): 36.2°; Angle between the anterior cranial base (Sella to Nasion) and the mandibular plane (Gonion to Menton) (SN/MP): 44.6°) (Table 1). The maxillary incisors showed labial inclination (angle between the long axis of the most protruded maxillary incisor and the Sella-Nasion (SN) line (U1 to SN): 112.1°; the distance between the tip of the upper incisor and Frankfort horizontal plane (U1 to FH): 120.6°; and interincisal angle: 110.9°) (Table 1). No discrepancy between centric occlusion and centric relation was observed, and the mandibular position was stable (Figure 5). No findings suggestive of temporomandibular disorder were observed.

**Table 1:** Cephalometric analysis of pre-treatment and post-treatment. SNA: Angle between the anterior cranial base (Sella to Nasion) and the NA (Nasion to point A) line; SNB: Angle between the anterior cranial base (Sella to Nasion) and the NB (Nasion to point B) line; ANB: Angle between NA (Nasion to point A) and NB (Nasion to point B) lines, FMA: Angle between the FH (Frankfort horizontal) plane (Porion to Orbitale) and the mandibular plane (Gonion to Menton); SN/MP:Angle between the anterior cranial base (Sella to Nasion) and the mandibular plane (Gonion to Menton); U1 to SN: angle between the long axis of the most protruded maxillary incisor and the Sella-Nasion (SN) line; U1 to FH: the distance between the tip of the upper incisor and Frankfort horizontal plane; IMPA: Incisor Mandibular plane angle is the angle formed between mandibular plane and the long axis of the mandibular incisor; FMIA: the angle formed between Frankfort horizontal plane and long axis of mandibular incisor; U1 to A-Pog: The upper incisor to A-pogonion; L1 to A-Pog: the linear measurement between the edge of the mandibular incisor and a line drawn from point A to pogonion; E line: a line from the pronasale to the pogonion

		Mean	Initial	Final
Skeletal pattern	SNA (°)	81.5	84.3	84.3
	SNB (°)	77.6	74.3	76.5
	ANB (°)	3.9	10	7.8
	Facial angle (°)	85.5	82.2	85
	Y-axis (°)	64	68.1	67.8
	FMA (°)	27.7	36.2	39.1
	SN/MP (°)	33.4	44.6	48.8
	Gonial angle (°)	121	127.6	132.7
Denture pattern	Occ. plane to SN (°)	13	19.6	27.4
	U1 to SN (°)	105	112.1	100.1
	U1 to FH (°)	109.8	120.6	109.7
	IMPA (°)	91.3	92.4	91.5
	FMIA (°)	61	51.4	49.4
	Interincisal angle (°)	124.1	110.9	119.7
	U1 to A-Pog (mm)	7.5	17.1	9.9
	L1 to A-Pog (mm)	4.4	3	7.1
Soft tissue	E-line : Upper Lip (mm)	1.8	15.6	26.2
	E-line : Lower Lip (mm)	2.9	-3	-0.8

Medical history

Port-wine stains were present bilaterally on the face along the distribution of the 1st and 2nd branches of the trigeminal nerve and over the left axilla, medial upper arm, and shoulder since birth. Two months after birth, the patient experienced convulsions on the right side of the body, and valproic acid administration was initiated. The last epileptic seizure occurred at the age of 4 years, and antiepileptic drugs were discontinued by self-judgement at approximately 12 years of age. At the age of 2 years, paresis appeared on the right side of the body; although the patient was able to walk by himself, he walked with a limp. On the first visit, the patient rarely used his right hand for daily activities. No motility problems were observed in the lower extremities. The patient had right hemianopia due to glaucoma and had undergone several ophthalmic surgeries under general anesthesia. A slight laterality in the development of the facial muscles was noted, and food sometimes leaked from the right side of the lips when eating. The patient had a history of multiple laser treatments under general anesthesia for hemangiomas of the upper lip since infancy, with limited improvement. The patient’s intelligence was borderline; however, verbal communication skills were good.

Treatment plan and progress

Orthognathic treatment using a bilateral sagittal split osteotomy procedure was proposed to correct the jaw deformities. An alternative treatment plan was considered; however, establishing satisfactory occlusion without surgery seemed difficult. Improvement of the labial inclination of the maxillary incisors by using space was planned. Thus, orthodontic treatment without tooth extraction was planned. Bands with buccal tubes were adapted to the first molars in both arches. A transpalatal arch was adapted to the upper arch, and a lingual arch was adapted to the lower arch to control the width of the dental arches and the torque of the molars. In addition, bands were adapted to the second molars in both arches to prevent displacement of bondable tubes in the operative field. Slotted 0.018” standard edgewise brackets were directly bonded to the teeth in both arches. The following sequence of arch wires was used for alignment and leveling: nickel-titanium, 0.014” and 0.016” and cobalt-chromium, 0.016”× 0.016” and 0.016”× 0.022”. An orthodontic anchorage system (OAS, ISA Advance; length, 6.0 mm; diameter, 2.0 mm; Platonjapan, Tokyo, Japan) was placed near the mid-palatal suture. Although this was a non-extraction case, OAS was used to prevent excessive intraoperative anterior displacement of the mandible due to mesial movement of the maxillary molars during space closure. To achieve maximum anchorage, the OAS was connected to the transpalatal arch using a ligature wire. Spaces were closed using a 0.016”× 0.022” stainless steel wire with Bull’s teardrop loops. During the preoperative orthodontic treatment, immediate plaster models were fabricated at the chairside to check the molar and canine widths, molar torque, and anterior overjet.

**Figure 6:**
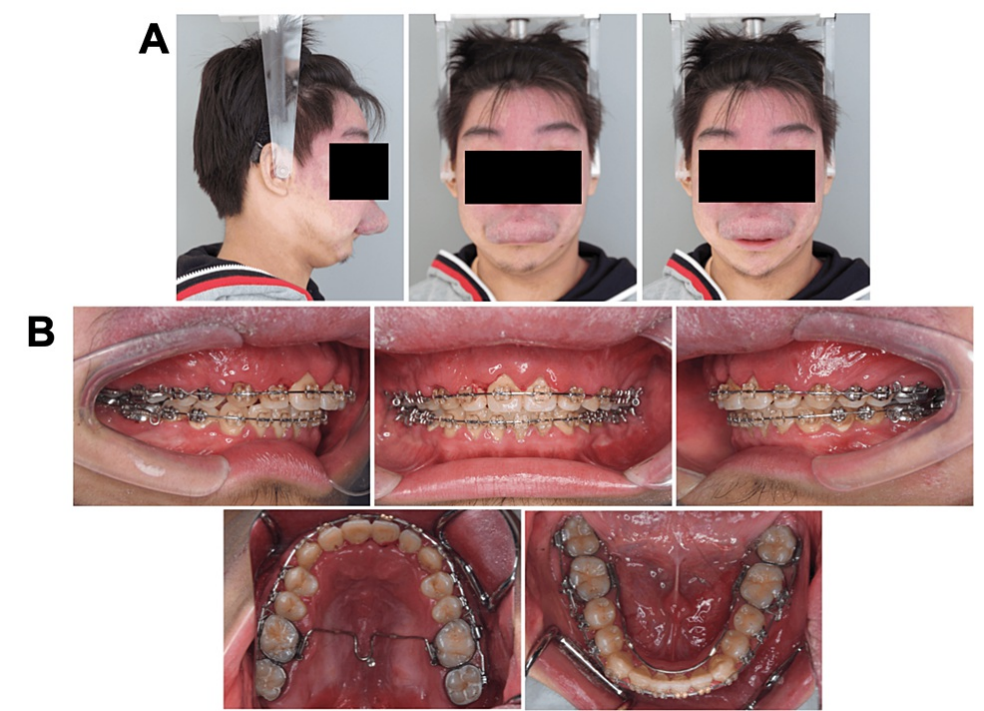
Presurgical evaluation. (A) Facial photographs. (B) Intraoral photographs.

**Figure 7:**
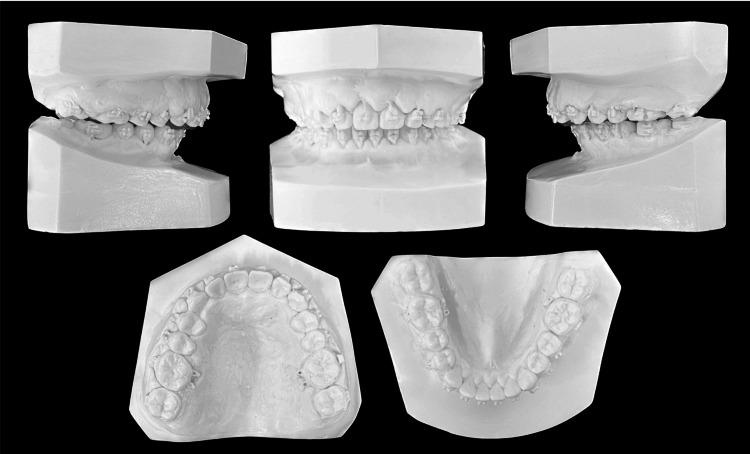
Presurgical evaluation. Plaster models.

**Figure 8:**
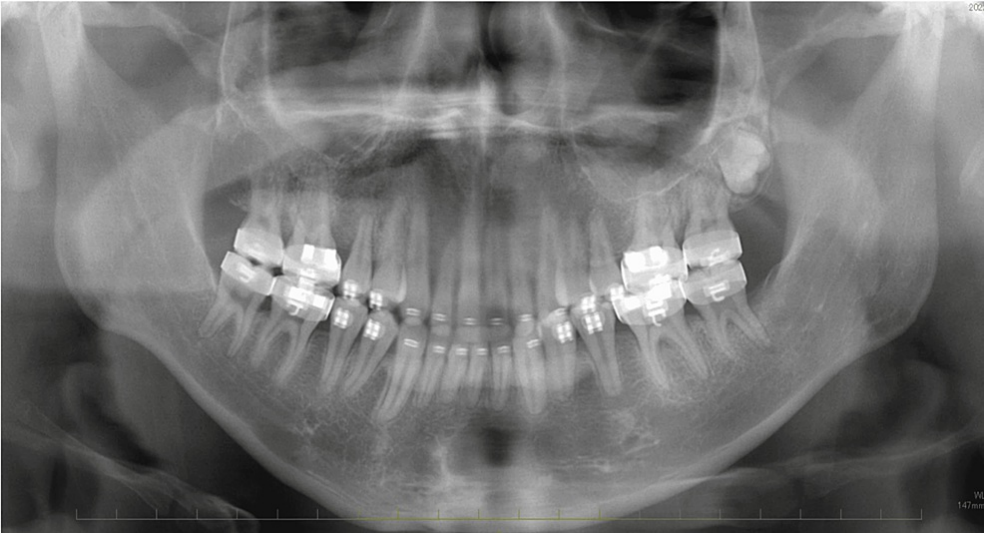
Presurgical evaluation. Panoramic radiograph.

**Figure 9:**
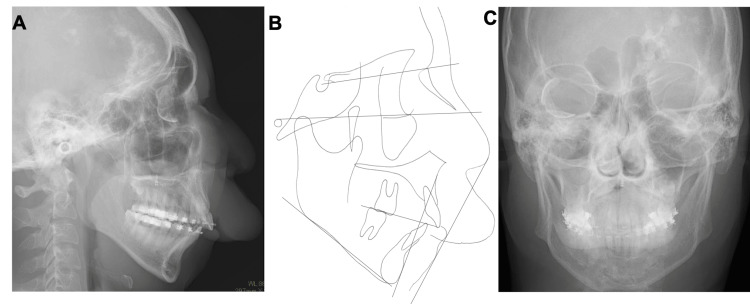
Presurgical evaluation. (A) Lateral cephalometric radiograph. (B) Original tracing of lateral view. (C) Frontal cephalometric radiograph.

**Figure 10:**
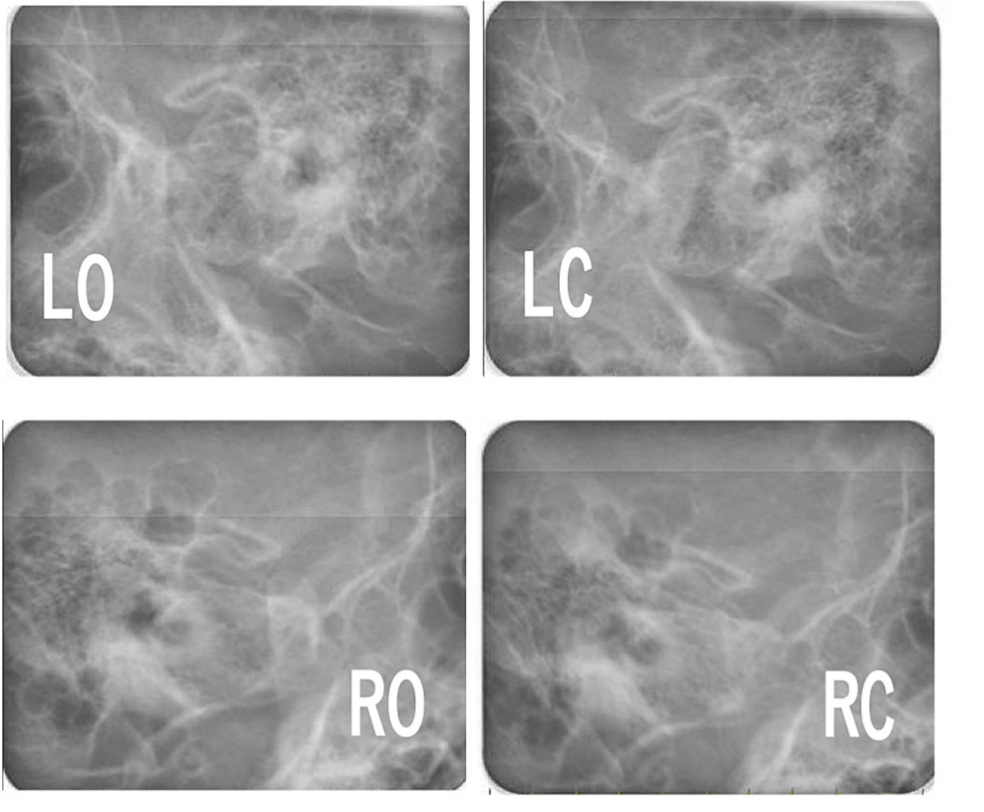
Presurgical evaluation. Schuller’s radiographs. LO, left open; LC, left close; RO, right open; RC, right close.

At the end of the preoperative orthodontic treatment, the labial inclination of the maxillary incisors improved. However, the upper-lip hypertrophy was exacerbated owing to hemangioma enlargement (Figure 6A). The interdental spaces in both arches were closed, and the coronal arch widths were harmonized (Figures 6B, 7). Maxillary gingival swelling was exacerbated due to hemangioma enlargement and poor oral hygiene. The mandibular left third molar was extracted to prepare for sagittal split ramus osteotomy (SSRO) (Figure 8). Deviation of the mandible to the right side remained unchanged (Figure 9). The condylar position and movement was not changed as well from the start of treatment (Figures 10).

**Figure 11:**
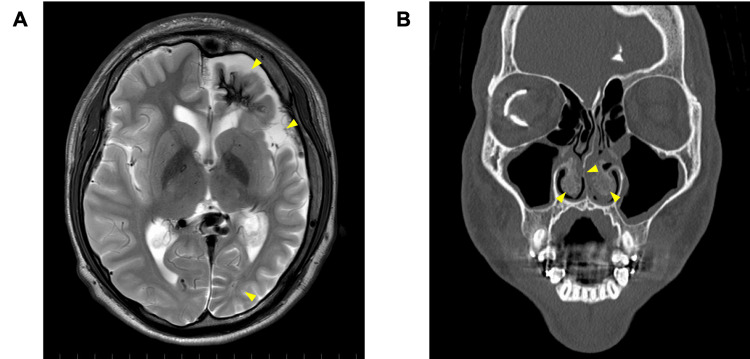
Presurgical evaluation. (A) Fat-suppressed T2 weighted magnetic resonance image. The arrowhead shows the atrophy of the left cerebral hemisphere which is typical symptom of SWS. (B) Computed tomography image. The arrowhead shows that the inferior turbinate was thickened and the nasal septum had deviated. This makes nasal intubation difficult.

During the preoperative evaluation, the patient’s blood pressure, pulse rate, and peripheral arterial oxygen saturation levels were normal. Magnetic resonance imaging in consultation with the Department of Neurosurgery revealed a hemangioma along the gyrus between the frontal and occipital lobes of the left hemisphere, atrophy of the left cerebral hemisphere, and calcification along the brain surface (Figure 11A). The dental anesthesiologist monitored the blood pressure and maintained normotension as a precaution during the perioperative period. Computed tomography revealed that the inferior turbinate was bilaterally bony and thickened, and the nasal septum had deviated to the right side (Figure 11B). In addition, redness due to the hemangioma was prominent around the inferior turbinate. Therefore, nasal intubation, which is commonly performed during orthognathic surgery, was predicted to be difficult. The Department of Otolaryngology suggested that nasal-septum correction and inferior-turbinate resection should be performed prior to nasal intubation. However, since preoperative orthodontic treatment had been performed for several years, submental intubation was performed for early termination [14]. Although tracheostomy was an option, it was not chosen because it was likely to be performed when the hemangioma was debulked by the department of plastic surgery after the completion of orthodontic treatment. Autologous blood was collected in advance to prepare for blood transfusions during surgery. Only mandibular surgery was planned to avoid the risk of abnormal bleeding from the maxillary hemangioma.

SSRO was performed with 6.2 and 3.4 mm of advancements on the right and left sides, respectively. In addition, a 2.9 mm leftward yaw rotation was performed to coincide the mandibular dental midline with the facial and maxillary dental midlines. The distal segment of the mandible was completely transected from the mesial segment, and the medial pterygoid muscle and stylomandibular ligament were detached. After removing the interfering parts of the distal and mesial fragments, a final splint was inserted, and intermaxillary fixation using ligature wires was performed. The bone junction was fixed using titanium plates (MatrixMANDIBLETM Plating System; Johnson & Johnson, USA). The surgery was completed after releasing the fixation and confirming that the mandible did not deviate from the targeted position.

Considering the risk of suffocation due to submandibular swelling caused by submental intubation, intermaxillary fixation was not performed on the day of the surgery, and intermaxillary fixation using 0.012” ligature wires was applied for 5 days from postoperative day 1. After releasing the intermaxillary fixation, the patient was instructed to use class II and midline elastics at all times, except during meals and tooth brushing, for three months. No temporomandibular joint disorders were observed after surgery. Paresthesia of the inferior alveolar nerve disappeared completely one month after surgery.

Seven months after surgery, all appliances were removed, and lingual retainers were bonded between the 1st premolars in both arches. The patient was instructed to wear Begg-type retainers daily at bedtime. 

Treatment outcome

**Figure 12:**
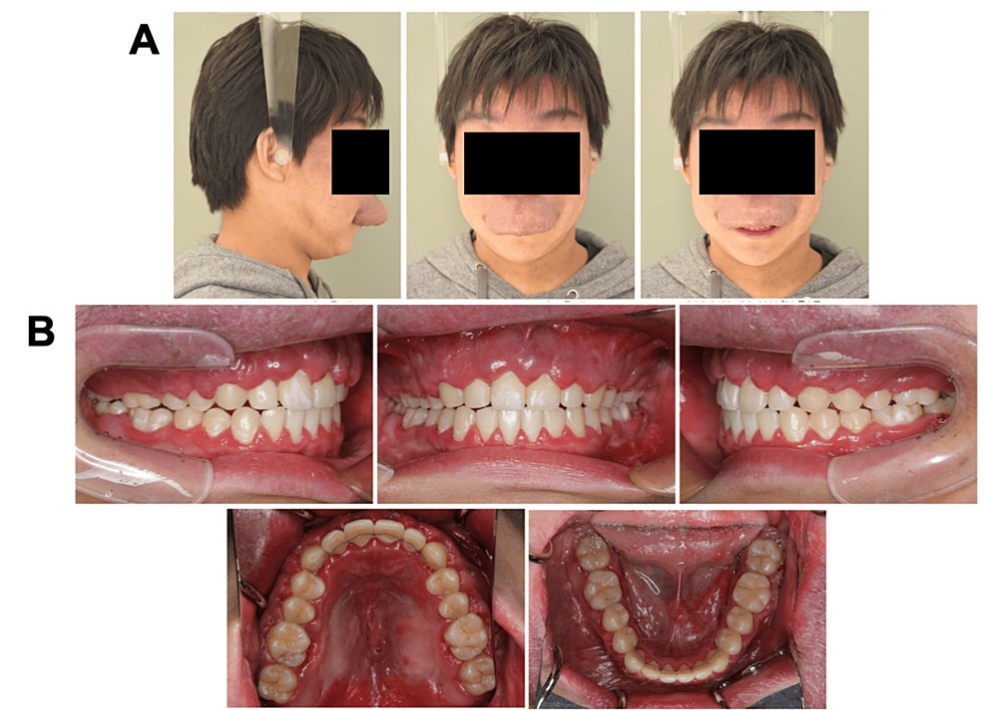
Post-treatment evaluation. (A) Facial photograph. (B) Intraoral photograph.

**Figure 13:**
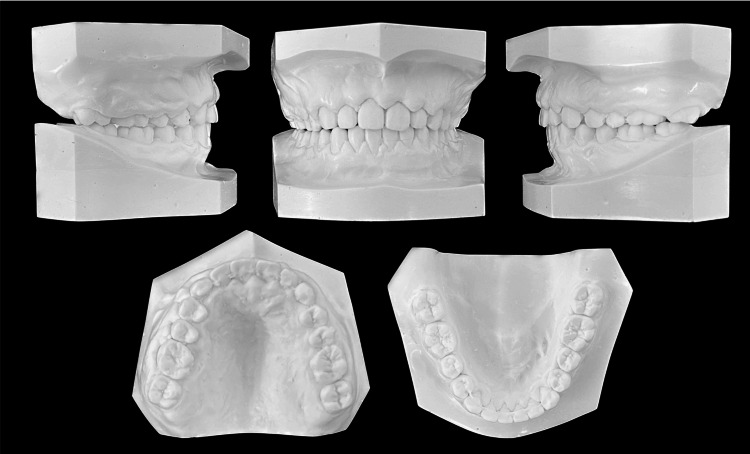
Post-treatment evaluation. Plaster cast model.

**Figure 14:**
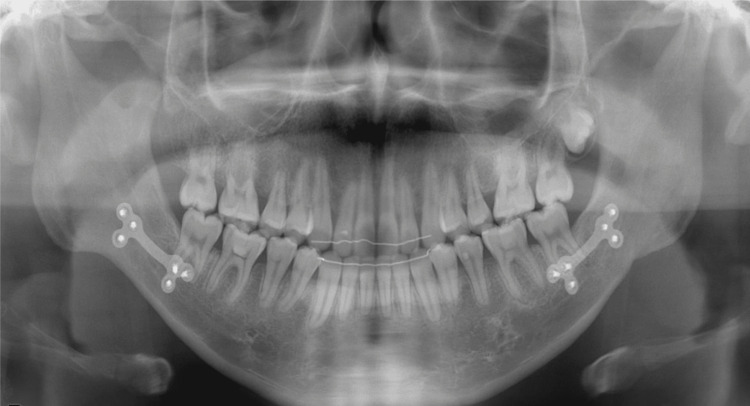
Post-treatment evaluation. Panoramic Radiograph.

**Figure 15:**
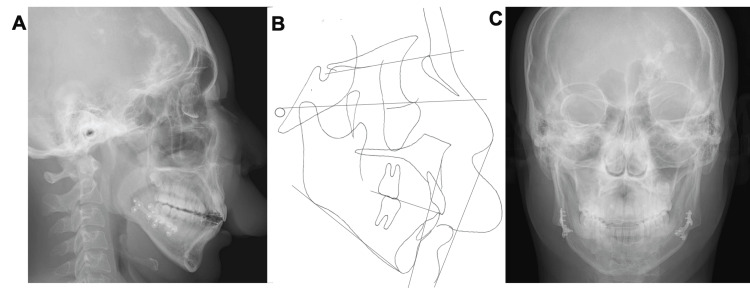
Post-treatment evaluation. (A) Lateral cephalometric radiograph. (B) Original tracing of lateral view. (C) Frontal cephalometric radiograph.

**Figure 16:**
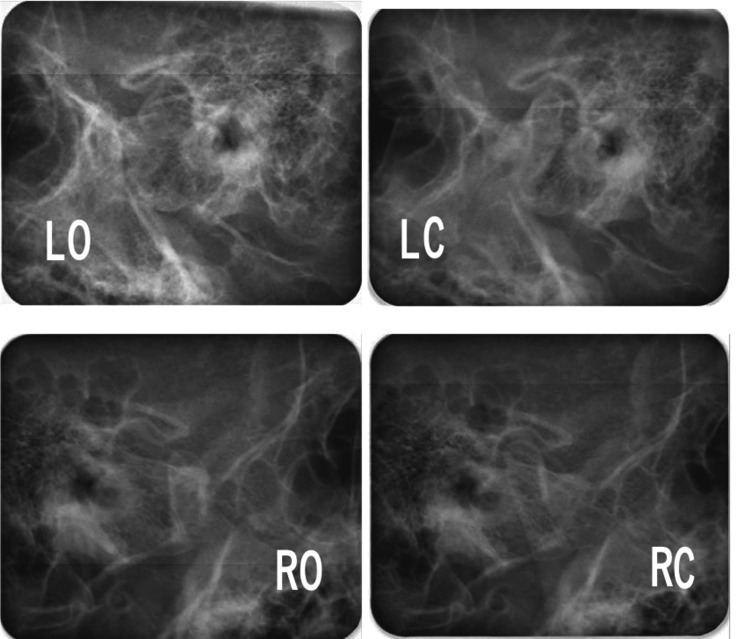
Post-treatment evaluation. Schuller’s Radiograph. LO, left open; LC, left close; RO, right open; RC, right close.

**Figure 17:**
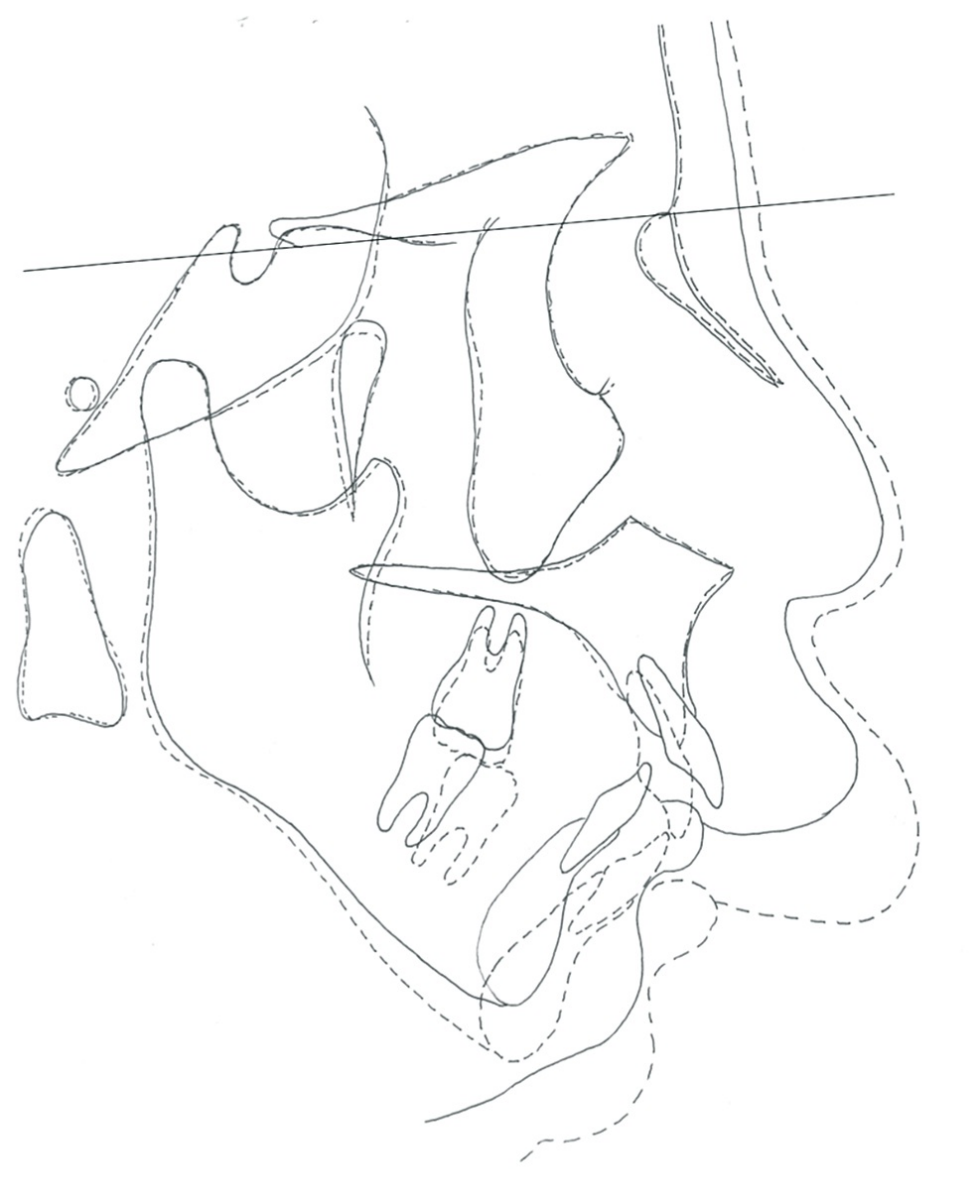
Post-treatment evaluation. Cephalometric superimposition: solid line, pretreatment; dotted line, post-treatment.

The postoperative facial photograph shows enhancement of the upper-lip hypertrophy due to the hemangioma. Although retrognathia improved due to the forward displacement of the mandible, the profile remained convex (Figure 12A). The maxillary and mandibular arches were well-aligned, and the deep overbite improved. The overjet was reduced from 13.9 mm to 1.5 mm, and bilateral angle class Ⅰ molar and canine relationships were established (Figures 12B, 13). Although the mandibular dental midline was not perfectly matched with that of the maxillary arch due to postoperative relapse, it improved compared to the initial state (Figures 14, 15C). Cephalometric analysis performed to confirm the improvement (Figure 15) revealed that angle SNA was maintained, and anterior displacement of the mandible increased angle SNB to 76.5°; however, angle ANB improved only to 7.8° (Table 1). The FMA and SN/MP increased to 39.1° and 48.8°, respectively, due to the clockwise rotation of the mandible. The positions of the mandibular condyles did not change, and the condylar path during the maximum open-close motion was stable (Figure 16). Cephalometric superimposition confirmed improvements in skeletal maxillary protrusion, labial inclination of the maxillary incisors, mandibular incisor extrusion, and the curve of Spee (Figure 17). The occlusion remained stable at the last follow-up. 

## Discussion

**Figure 18:**
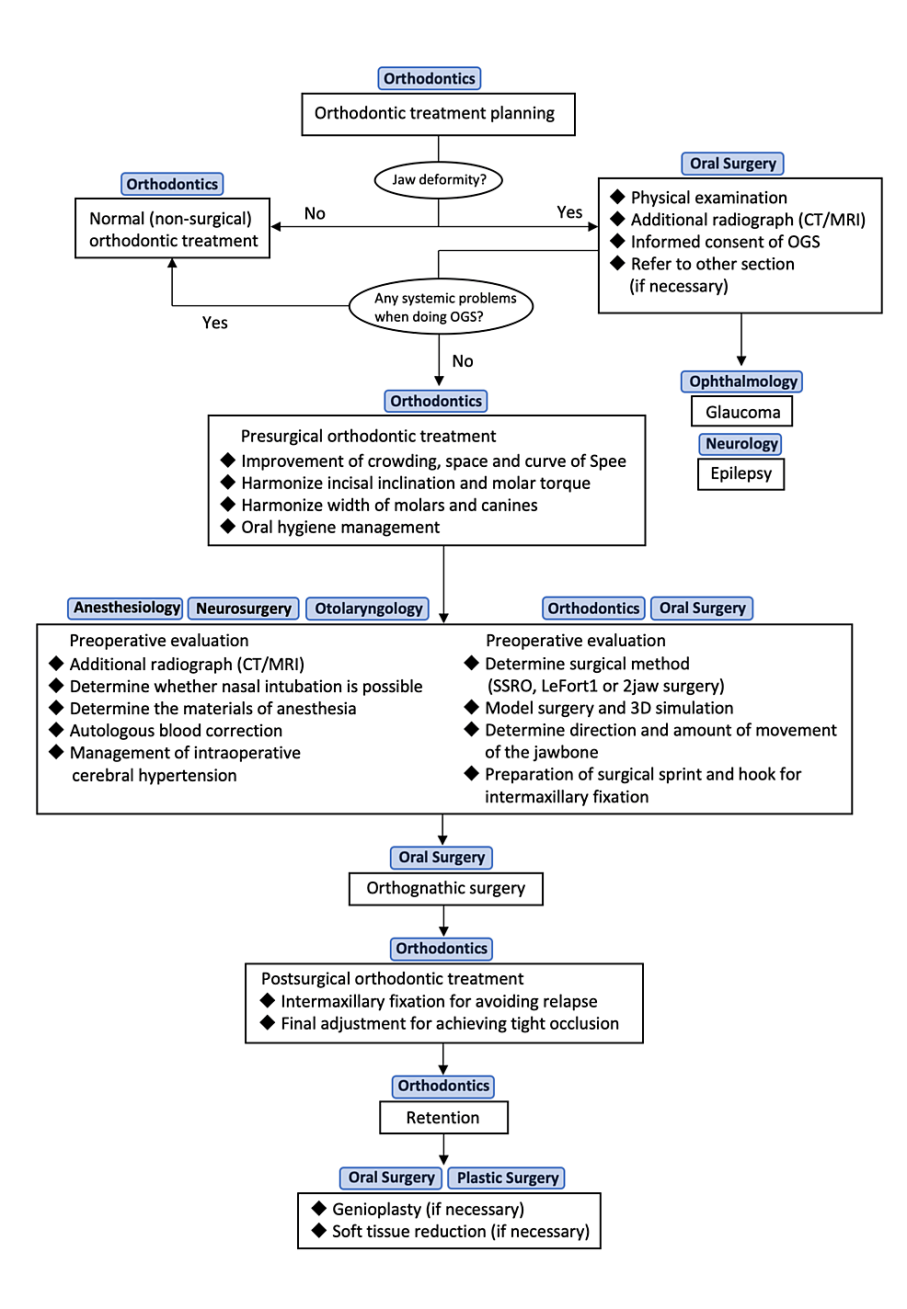
Treatment algorithm for SWS patients. OGS, Orthognathic Surgery; SSRO, Sagittal Split Ramus Osteotomy.

SWS is a congenital disease characterized by the development of hemangioma [1]. Previous reports have suggested that surgical procedures should be avoided in patients with SWS because of the risk of abnormal bleeding [11,15]. In another report, surgical orthodontic treatment was performed for SWS patients, but there were no restrictions regarding anesthesia and surgical technique because the port-wine stain and hemangioma were unilateral and limited in location [10]. In this case, no hemangioma was observed in the mandibular region; therefore, surgical orthodontic treatment was selected. However, the choice of surgical procedures was limited. Based on the results of the cephalometric analysis, segmental osteotomy of the maxilla was indicated to reposition the anterior segment of the maxilla posteriorly [16]. However, because of the presence of a hemangioma around the maxilla, only SSRO was performed. When treating SWS patients, who may have some risks and restrictions on treatment, as in this case, it is necessary to collaborate closely with various clinical departments for diagnosis and treatment (Figure 18).

In this case, preoperative orthodontic treatment required approximately 5 years. The delay was attributed to three main reasons. The first factor was the frequency of patient visits. The patient was often admitted to a distant hospital for treatment of glaucoma and became ill. Therefore, the gap between the visits to our hospital was often 2-3 months. The second factor was the velocity of tooth movement. Bony overgrowth has been reported in 22% of patients with SWS, with the maxilla (83.0%) being the most frequently hypertrophied [7], and histopathological examination showed intraosseous vascular malformation [7]. This bony abnormality in the maxilla likely caused the teeth to move slower than usual. The third factor was the personnel shifts in each department. As the treatment of patients with congenital diseases requires specialized techniques and close collaboration between several departments, factors other than medical parameters may affect the progress of treatment.

Generally, nasal intubation is performed during SSRO because the orthodontist needs to check the occlusion during surgery. However, nasal intubation was avoided in this patient because of the angiomas in the nasal cavity and the thickening of the bilateral inferior turbinates. Therefore, submental intubation was selected for airway management [17].

Anticlockwise rotation of the mandible with anterior displacement is associated with relapse. This relapse is thought to be caused by an increase in the length of the posterior ramus and stretching of the masseter and internal pterygoid muscles. In this case, because the mandible was rotated clockwise, anteroposterior relapse was not observed. However, mandibular deviation to the right gradually relapsed. Schuller’s radiographic view revealed that the position of condyles with respect to the mandibular fossa was acceptable; thus, condylar sag was unlikely [18-20]. The mandible was likely pulled to the right due to stretching of the skin, ligaments, and masticatory muscles. To correct this relapse, the patient was instructed to use midline intermaxillary elastics. Although he made a great effort, the patient was unable to apply the elastics by himself due to hemiplegia and needed the help of his parents. Therefore, the duration of use of intermaxillary elastics was limited, and the midline did not perfectly coincide.

The protruded upper lip, which was one of the patient’s chief complaints, was exacerbated during orthodontic treatment. Soft-tissue hypertrophy occurs in 55% of patients with SWS, and the lips are the most frequently hypertrophied (81.0%) [7]. In the future, plastic surgery is scheduled for volume reduction of the upper lip, excision of port-wine stains, and skin grafting [15].

## Conclusions

Surgical procedures in patients with SWS are associated with various risks that may restrict the choice of anesthetic and surgical modalities. However, orthognathic surgery can be performed after carefully evaluating the sites and sizes of the hemangiomas, even in patients with SWS. 
